# Identifying Barriers to OUD Treatment Linkage From the Emergency Department to the Community

**DOI:** 10.1001/jamanetworkopen.2023.12683

**Published:** 2023-05-01

**Authors:** Susan L. Calcaterra, Marlene Martin, Honora Englander

**Affiliations:** Department of Medicine, Division of General Internal Medicine and Division of Hospital Medicine, University of Colorado, Aurora; Department of Medicine, Division of Hospital Medicine, University of California San Francisco and San Francisco General Hospital, San Francisco; Department of Medicine, Section of Addiction Medicine and Division of Hospital Medicine, Oregon Health and Science University, Portland

Associated with illicitly manufactured fentanyl and its analogues, the current opioid overdose epidemic is the largest recorded in US history.^1^ Emergency departments (ED) are essential sites of care for people with opioid use disorder (OUD) and present an opportunity to initiate medications for OUD, including buprenorphine. ED-initiated buprenorphine combined with a follow-up appointment and buprenorphine prescription increases OUD treatment engagement and decreases self-reported illicit opioid use.^2^ A recent study by Hawk et al^3^ of ED practitioners identified a lack of training or experience with buprenorphine, concerns about availability of outpatient follow-up, and competing needs and priorities for ED resources as barriers to ED-initiated buprenorphine. However, ED practitioners also reported a willingness to initiate buprenorphine with education and training and with availability of local protocols to initiate buprenorphine and pathways to link patients to ongoing OUD care.^3^ Addressing and reducing these barriers is imperative to improve ED-initiated buprenorphine and OUD treatment referral. Although ED-initiated buprenorphine is the first step to support patients experiencing opioid withdrawal and to treat OUD, ongoing OUD treatment engagement is necessary to reduce overdose mortality.^4^ Thus, building referral pathways between local ED and community OUD treatment practitioners is crucial to ensure that people initiated on buprenorphine in the ED can continue to receive buprenorphine to support their recovery.

In this formative evaluation, Sue and colleagues^5^ explored perspectives of practitioners and staff working in community-based programs who could potentially continue providing OUD treatment for patients initiated on buprenorphine in local EDs. The study results informed implementation activities related to Project ED HEALTH, a hybrid type 3 effectiveness-implementation study that aimed to measure the outcomes associated with implementation facilitation to promote ED-initiated buprenorphine for OUD.^6^ The authors conducted 14 focus groups with a total of 103 participants from a variety of health care settings across 4 large urban areas. OUD treatment settings included primary care centers, office-based buprenorphine programs, opioid treatment programs, programs that offered medically managed withdrawal (eg, detoxification), intensive outpatient programming, and programs that offered services for people with co-occurring mental health and substance use disorders. Emergent themes included: (1) negative attitudes toward buprenorphine and (2) variable attitudes toward ED-initiated buprenorphine, (3) limited treatment capacity to add new patients, (4) payment and regulatory barriers that disincentivized buprenorphine provision, (5) need for more highly trained substance use treatment practitioners, (6) need for improved communication across health care systems, (7) and need to address poverty, homelessness, and other social determinants of health to support recovery from substance use.

In the complementary Sue et al^5^ and Hawk et al^3^ studies, community-based addiction treatment practitioners and ED practitioners highlighted the need for expanded OUD treatment capacity in the community. A multipronged approach is essential to expand access to buprenorphine across multiple health care settings and to normalize buprenorphine prescribing among all practitioners. Some steps at the federal level have already been taken to reach these goals.

In response to the COVID-19 pandemic in March 2020, the federal government enacted 2 major changes that increased access to buprenorphine. First, the Substance Abuse and Mental Health Administration (SAMHSA) updated 42 CFR Part 8^7^ to eliminate the in-person requirement for telehealth-delivered buprenorphine. Second, SAMHSA and the Drug Enforcement Administration (DEA) allowed buprenorphine appointments to be delivered by telephone (ie, audio-only, not limited to a video encounter). At the time, these 2 regulatory changes intended to protect practitioners and patients from contracting and spreading COVID-19. They had the additional benefit of expanding buprenorphine access to people living in rural areas, to marginalized populations, to people who lacked reliable transportation, and to people who were immobile. Subsequent studies demonstrated that these regulatory changes resulted in improved OUD treatment retention, greater patient satisfaction due to convenience, and stronger trust between patients and practitioners due to sustained contact with their practitioner.^8^

Unfortunately, despite these benefits, the DEA and the Department of Health and Human Services recently proposed to reinstate the requirement for an in-person examination for telehealth patients who remain on buprenorphine for longer than 30 days.^9^ If the proposed amendments are codified into law, buprenorphine access will decrease, and people living in rural areas and underserved communities will likely bear the burden of this proposed change. The change would magnify the barriers identified in the study by Sue et al,^5^ as community treatment centers already face limited treatment capacity and difficulties addressing sociostructural marginalization.^5^

A third step toward reducing regulatory barriers to buprenorphine prescribing was the passage of the “Mainstreaming Addiction Treatment Act,”^10^ signed into law by President Biden in December 2022, which eliminated the DEA X-Waiver. Although many advocates hailed these 3 federal changes, others remained skeptical they would substantially impact buprenorphine prescribing because barriers to buprenorphine provision go far beyond the elimination of the X-Waiver and expanded access to buprenorphine via telehealth, as evidenced by the stigma toward buprenorphine reported by Sue et al.^5^

To address the barriers highlighted by clinicians and staff in community settings in the study by Sue et al,^5^ we need multiple targeted implementation strategies. First, enforcement of parity requirements for insurance coverage for addiction treatment could increase reimbursement of services to incentivize buprenorphine provision, support the cost of recruiting and retaining substance use treatment practitioners in community settings, and cover the cost of peer navigators to facilitate treatment linkage from the ED to the community. Health care systems and their leaders should prioritize OUD treatment provision by establishing quality metrics or clinician incentives tied to the provision of evidence-based OUD treatment, not only in the ED, but in the inpatient and outpatient settings, to encourage practice change.

Next, continuation and expansion of telehealth for buprenorphine initiation and follow-up narrows a treatment gap for many people who lack access to a local buprenorphine prescriber.^8^ Given the lethality of the illicit drug supply, further expanding buprenorphine treatment access through low-threshold programs, such as same day or walk in-treatment with provision of harm reduction education, supplies, and services must be incorporated into existing programs. Such changes could be implemented by community health care system leaders and clinicians, local public health departments, and community activists.

Finally, mandating addiction education as a training requirement for medical students and residents, advanced practice clinicians, and pharmacists could increase the number of practitioners who feel comfortable prescribing buprenorphine as part of their routine clinical practice. As more clinicians integrate OUD treatment into their practice, it will normalize this care while simultaneously reducing stigma, a major barrier for clinician uptake of buprenorphine prescribing. With increased education regarding the life-saving benefits of buprenorphine and its safety, there would likely be greater uptake of medication-first strategies where buprenorphine provision is not coupled with punitive restrictions and contingencies, which leads to people leaving care and returning to illicit opioid use.

Sue et al^5^ highlight barriers reported by community-based OUD treatment practitioners to accepting patients who received ED-initiated buprenorphine while simultaneously providing actionable solutions to address these barriers. The unifying theme across ED clinicians^3^ and community-based treatment practitioners^5^ is the concern for continued treatment access once initiated on buprenorphine. Although participants in both studies practiced in urban settings, it is likely that the barriers reported here are magnified in rural and under resourced communities. Strategies aimed at addressing concerns both reported by ED clinicians and community-based clinicians are imperative to close an OUD treatment gap and save lives.
